# The New Anatomy

**Published:** 1899-12

**Authors:** 


### The New Anatomy.

*Practical Medicine* for May has collected the following instances of the newer (literary) anatomy:

The murderers have discovered some astonishing vulnerable parts of the human anatomy of late. From a paper this morning we learn that a Georgia colonel was "shot in the ticket office;" the other day a man was fatally shot "through his door," and not long ago another received a fatal wound "in his window."—*N. Y. Commercial Advertiser.*

He kissed her passionately upon her reappearance.—*Jefferson Souvenir.*

She whipped him upon his return.—*Hawkeye.*

She seated herself upon his entering.—*Akia Democrat.*

We thought she sat down upon her being asked.—*Saturday Gossip.*

She fainted upon his departure.—*Lynn Union.*

He kicked the tramp upon his sitting down.—*American Pharmacist.*

We feel compelled to refer again to the poor woman who was shot in the oil regions some time ago.—*Med. World.*

The fact of the woman being accidentally shot in the waterworks, or the man injured upon the long bridge.—*Colorado Med. Journal.*

And why not drop a tear for the man who was fatally stabbed in the rotunda, and for him who was kicked on the highway?—*Lancet Clinic.*

